# Childhood Hypertension: A Retrospective Analysis of Causes, Treatments, and Complications

**DOI:** 10.3390/children11101234

**Published:** 2024-10-14

**Authors:** Mohamed S. Al Riyami, Aisha Al Shuaibi, Suad Al Jardani, Asma Elfar, Anisa Al Maskari, Badria Al Gaithi, Sulaiman Al Saidi, Naifain Al Kalbani

**Affiliations:** 1 Pediatric Nephrology Unit, Department of Child Healthy, Royal Hospital, Muscat P.O. Box 1331, Oman; aisha.alshuaibi@moh.gov.om (A.A.S.); su3ad118@gmail.com (S.A.J.); asmaa.ahmed3@moh.gov.om (A.E.); anisaalmaskari@yahoo.com (A.A.M.); badria.al-ghaithi@moh.gov.om (B.A.G.); suleiman.alsaidi@moh.gov.om (S.A.S.); naifain.alkalbani@moh.gov.om (N.A.K.); 2 Pediatric Residency Training Program, Oman Medical Specialty Board, Muscat P.O. Box 1422, Oman

**Keywords:** childhood hypertension, Omani children, primary hypertension, secondary hypertension

## Abstract

Background: Hypertension is prevalent in the pediatric population, with estimated rates between 2% and 5%, and its incidence is rising globally. This study offers a single-center analysis of hypertension in children. Methods: a retrospective chart review was conducted involving children aged 1 month to 13 years diagnosed with hypertension. Results: The study included a total of 129 children. Secondary hypertension was identified in 103 patients (79.8%), while primary hypertension was noted in 26 patients (20.2%). Primary hypertension was more common among pre-teen children (50.0%), whereas secondary hypertension predominantly affected those aged 1 to 5 years. Renal parenchymal disease emerged as the most frequent etiology of secondary hypertension, followed by endocrine disorders and vascular issues. No significant correlation was found between hypertension and obesity. The primary complications associated with hypertension in these children were cardiovascular, followed by neurological issues. A small proportion (14.7%) managed their hypertension solely through lifestyle modifications, while the majority required additional antihypertensive medications. At the final follow-up, 50% of the children demonstrated improved blood pressure readings. Conclusion: The findings indicate a higher prevalence of secondary hypertension compared to primary hypertension among the studied population. This study underscores the necessity for heightened awareness among pediatricians regarding the early identification and management of hypertension. Larger population-based studies are warranted to further investigate the prevalence, causes, and outcomes of hypertension in this region.

## 1. Introduction

Hypertension is a prevalent chronic condition frequently encountered by pediatric healthcare providers [1]. The global prevalence of hypertension in children is rising, with estimates ranging from 1.6% to 3.5%. Furthermore, an additional 2.2% to 9.4% of children exhibit blood pressure measurements that exceed the accepted normal range [2,3]. Childhood hypertension is a predictor of hypertension in adulthood and serves as an early indicator of cardiovascular disease [4,5,6]. Several factors contribute to the increasing prevalence of hypertension in this population, notably obesity, which is associated with a higher intake of high-calorie and salty foods alongside decreased physical activity [7]. Given that hypertension is often asymptomatic, end-organ damage may already be present by the time primary hypertension is diagnosed [8,9].

Indicators of organ damage, such as left ventricular hypertrophy, microalbuminuria, and impaired cognitive function, have been documented in hypertensive children [8]. Thus, the prompt detection and management of childhood hypertension are crucial to mitigating related health complications and mortality [8].

Secondary hypertension is more prevalent among infants and preschool-aged children, whereas primary hypertension is more commonly diagnosed in older children and adolescents [10]. Current guidelines recommend monitoring blood pressure in all children aged three years and older; however, routine assessment is not consistently performed in various healthcare settings [8].

Challenges to regular blood pressure measurement during routine health visits include difficulties in keeping children calm and ensuring they are adequately rested for accurate readings. Additionally, abnormal blood pressure readings may go unrecognized, as normal values vary with age and sex in children [1]. Unlike adults, who have a standardized diagnostic threshold (e.g., 140/90 mm Hg), no universal threshold exists for diagnosing hypertension in children [1]. These complexities in interpreting blood pressure values contribute to the challenge of diagnosing hypertension in the pediatric population, often resulting in underdiagnosis [1].

The American Academy of Pediatrics recommends diagnosing hypertension in children under 13 years if their blood pressure measurements exceed the 95th percentile on three separate occasions, adjusted for age, gender, and height [11]. Despite this, there is a paucity of studies investigating the diagnosis and management of pediatric hypertension [11]. Data regarding childhood hypertension are notably lacking in Oman and other Gulf countries. In our region, there is a high rate of consanguinity and inherited renal diseases, in addition to other environmental and healthcare-related factors. These regional factors may uniquely shape the prevalence, causes, and outcomes of childhood hypertension. Understanding these regional differences can inform global strategies for hypertension management in pediatric populations, particularly in regions with similar sociodemographic and environmental conditions. Therefore, this study aims to elucidate the prevalent causes, complications, and treatment outcomes among children aged 1 month to 13 years diagnosed with hypertension.

## 2. Methods

### 2.1. Patient Population

This study encompassed all children aged 1 month to 13 years diagnosed with hypertension who were evaluated in the pediatric nephrology clinic or admitted to the pediatric wards at the Royal Hospital between January 2017 and December 2021. The Royal Hospital, the largest tertiary care center in Oman, provides comprehensive medical services and receives referrals from all regions of the country. Participants were referred to pediatric nephrology following the detection of elevated blood pressure, identified in both outpatient and inpatient settings within the hospital, as well as in regional hospitals.

Children diagnosed with transient hypertension were excluded from this analysis. Blood pressure measurements were conducted using the oscillometric technique on the upper limbs, utilizing cuffs of an appropriate size. Hypertension was defined in accordance with the 2017 American Academy of Pediatrics (AAP) guidelines, which require that the average of three consecutive systolic and/or diastolic readings meet or exceed the 95th percentile adjusted for age, sex, and height [11]. For 13-year-old children, hypertension is classified as having a blood pressure reading of 130/80 mm Hg or higher, in line with the 2017 AAP guidelines. For infants under one year of age, hypertension was defined as blood pressure readings exceeding the 95th percentile on three separate occasions [11].

Primary hypertension is defined as elevated blood pressure with no identifiable cause, whereas secondary hypertension is characterized by high blood pressure that arises from an underlying medical condition [5].

Prematurity was defined as a gestational age of less than 37 weeks.

The WHO growth charts for weight and height were employed to establish criteria for underweight, overweight, and obesity as follows: for children aged 5 to 13 years, underweight is defined as a body mass index (BMI)-for-age below the 3rd percentile, overweight is classified as a BMI-for-age above the 85th percentile, and obesity is defined as a BMI-for-age exceeding the 97th percentile [12].

Children diagnosed with hypertension underwent comprehensive assessments to exclude secondary causes, identify other cardiovascular risk factors, and evaluate for end-organ damage. This evaluation included urine tests, renal function assessments, electrolyte analysis, fasting blood glucose measurements, lipid profiling, and the measurement of renin and aldosterone levels. Imaging studies were also conducted, including ultrasounds of the kidneys, ureters, and bladder (KUB), with Doppler evaluations of renal vessels, as well as echocardiography. For those identified with stage II hypertension and lacking a clear underlying cause from the initial evaluation, further investigations were undertaken, including plasma metanephrine testing and either CT or MR angiography. In specific cases, conventional angiography was performed to evaluate for renovascular disease.

Data were extracted from the hospital’s electronic medical record system (Al Shifa) and entered into EpiData software. The collected data included patient demographics, laboratory and radiological investigations, diagnoses, treatments, and outcomes.

Approval for this study was granted by the Royal Hospital Ethical Committee.

### 2.2. **Statistical Analysis**

The data were initially entered into EpiData software (Version 4.6, released by The EpiData Association, Odense, Denmark), and subsequent analyses were conducted using SPSS Statistics for Windows, Version 20.0. Parametric *t*-tests were employed to compare groups for continuous variables, while Chi-square tests were utilized for comparing categorical variables. Kernel density curves were generated to visually represent the distribution of the age of hypertension diagnosis. A significance level of *p* < 0.05 was considered statistically significant.

## 3. Results

In the defined study period, a total of 159 patients were identified through the ALSHIFA system using the term “Hypertension”. Of these, 30 patients were excluded because they did not meet the inclusion criteria: 6 patients had pulmonary hypertension, 3 had intracranial hypertension, 10 had high blood pressure readings during the neonatal period, and 11 had transient systemic hypertension during acute illness that resolved without intervention on follow-up. Among the remaining 129 eligible children (see Table 1), 103 (79.8%) were found to have a secondary cause for their hypertension, while 26 (20.2%) had no identified cause (primary hypertension).

The demographic characteristics of the patients are detailed in Table 1. Of the total, 73 (56.6%) were male. A positive family history of hypertension was noted in 15 (57.7%) children with primary hypertension and in 50 (48.5%) of those with secondary hypertension. Additionally, 77 patients (60.2%) with hypertension were born to consanguineous parents. Among all children with hypertension, 27 (20.9%) were born prematurely.

**Figure 1:**
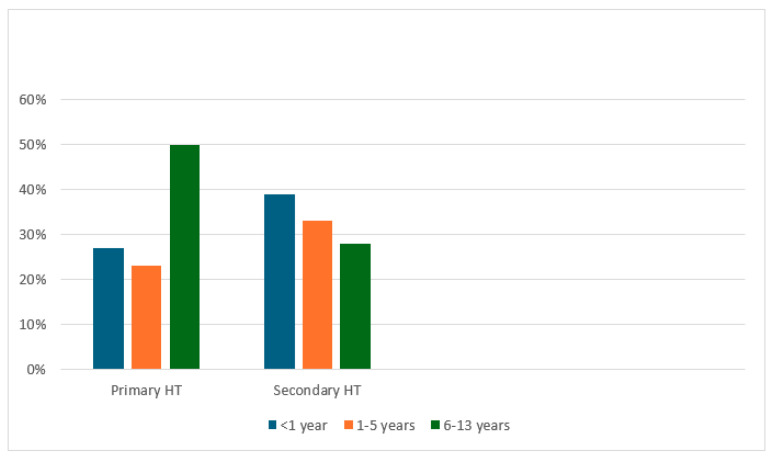
Primary vs. secondary hypertension by age groups. Abbreviation: HT, hypertension.

Primary hypertension was more prevalent among pre-teen children (50.0%), whereas a higher proportion (72%) of cases with secondary hypertension were aged below 5 years (Figure 1).

The etiology of hypertension is elaborated in Table 2. Renal parenchymal disease emerged as the predominant cause of secondary hypertension across all age groups, comprising 78 (60.5%) cases. This category includes conditions such as polycystic kidney disease (including autosomal recessive polycystic kidney disease and nephronophthisis), congenital anomalies of the kidney and urinary tract (CAKUT), steroid-resistant nephrotic syndrome (SRNS), glomerulonephritis, and other specified conditions, as listed in Table 2. Among children diagnosed with renal parenchymal disease, 39 (50%) were undergoing dialysis, and 6 had received kidney transplantation.

The second most common cause of hypertension in our patients was endocrine disorders, accounting for 11 (9.7%) cases. Among these, seven children were diagnosed with apparent mineralocorticoid excess (AME), two with hyperthyroidism, and two with Cushing syndrome. This is followed by renovascular disease in nine children (8.7%), including six with renal artery stenosis (RAS), one with both RAS and coarctation of the aorta, one with coarctation of the aorta alone, and another child with renal vein thrombosis. Other causes of hypertension include respiratory factors such as obstructive sleep apnea and medication-induced hypertension from steroids, each observed in two (1.94%) patients. Central nervous system (CNS) involvement contributed to hypertension in one (0.97%) patient.

**Figure 2:**
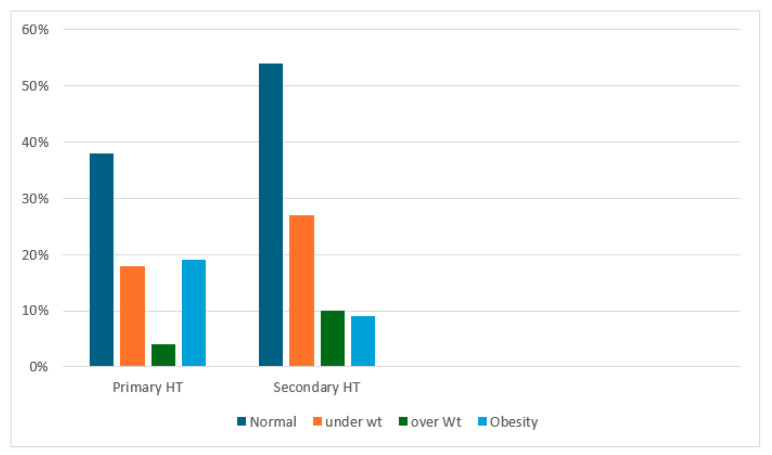
Association between hypertension etiology and weight. Abbreviation: HT, hypertension; Wt, weight.

Regarding obesity, it was found in five (19%) children with primary hypertension, while it was present in nine (8.7%) children with secondary hypertension (Figure 2).

Among children diagnosed with primary hypertension, 12 (46%) did not require any medications, while 7 (27%) were treated with one medication and a similar number with a combination of two medications. In contrast, antihypertensive medications were utilized in the majority of cases with secondary hypertension (Table 3). Approximately one-third of children with secondary hypertension required a combination of more than three medications to manage their blood pressure. A total of 14 (13.6%) patients with secondary hypertension required surgical intervention to control their blood pressure (Table 3).

The complications of hypertension in this cohort are summarized in Table 3. One of the most common reasons for hospital admission was hypertensive urgency in 57 (44%) children, significantly higher in secondary hypertension patients (53.4%) compared to those with primary hypertension (8%), with a *p*-value of 0.001. Cardiovascular complications—such as left ventricular hypertrophy or cardiomyopathy—were observed in 53 (41%) children and were significantly more prevalent in those with secondary hypertension (48 (47%)) compared to primary hypertension (5 (19.2%)), with a *p*-value of 0.019. Central nervous system complications were observed in 17 (16.5%) children, all of whom had secondary hypertension, with a *p*-value of 0.030. Fifteen patients (14.6%) presented with posterior reversible encephalopathy syndrome (PRES), and two (1.94%) had intracranial hemorrhage. At the last follow-up, blood pressure readings showed improvement in 50 (39%) of the cases compared to the readings at diagnosis.

## 4. Discussion

To the best of our knowledge, this study represents the first investigation into hypertension among children in Oman. We enrolled 129 children diagnosed with hypertension over a span of five years. Within our cohort, the majority (80%) were diagnosed with secondary hypertension, while 26 (20%) were diagnosed with primary hypertension. This distribution mirrors findings from Uhari et al., where 82% of children had secondary hypertension and 18% had primary hypertension [13]. Another retrospective cross-sectional study involving 231 hypertensive patients aged one to twenty years reported that 50.6% had primary hypertension and 49.4% had secondary hypertension [10], consistent with the findings of a multicenter clinical trial conducted by Flynn J et al. [14]. Our study focused on patients referred to our center for hypertension, with a significant number presenting with chronic kidney disease, which likely contributes to the relatively lower incidence of primary hypertension observed.

In our study, consanguinity was prevalent in both groups, with 64 (62%) of children with secondary hypertension and 13 (50%) of children with primary hypertension reporting consanguineous marriages. This trend aligns with the high rates of consanguinity observed in Oman and other Middle Eastern countries [15,16]. Similarly, a positive family history of hypertension was prevalent in both groups, with 51 (50%) and 15 (58%) children among those with secondary and primary hypertension, respectively. This prevalence can be attributed to a high rate of hereditary renal disease causing chronic kidney disease (CKD), leading to secondary hypertension affecting multiple family members. In a previous study, family history was present in only 27 cases (11.7%) and was more common in primary hypertension (20 cases, 20.6%) compared to secondary hypertension [10]. It is well established that genetic factors play a significant role in hypertension pathogenesis, as primary hypertension is frequently observed among parents and siblings and can also manifest among identical twins if one of them develops hypertension [17,18].

In our cohort, primary hypertension is predominantly observed in children aged 5–13 years, whereas preschool children are more likely to have secondary hypertension. This observation is consistent with several previous studies [1,8,14]. Monesha G et al. reported that primary hypertension is more prevalent in children older than 6 years of age [1]. Uysal et al. indicated that secondary hypertension is more common among younger children [10], while Flayen et al. found that secondary hypertension is more frequent in children below 6 years of age [14]. These collective findings underscore the importance of comprehensive evaluations for younger children with hypertension to exclude secondary causes.

In our cohort, hypertension was more prevalent in boys (56.6%) compared to girls (43.4%). This observation aligns with previous findings [1]. Among children with primary hypertension, 69% were boys, whereas among those with secondary hypertension, 53.3% were boys. In the adult population, primary hypertension is more commonly diagnosed in men compared to women [19].

In our cohort, 27 children (21.1%) were born prematurely, defined as less than 37 weeks of gestation, with 21 children (77.8%) developing secondary hypertension and 6 (22.2%) developing primary hypertension. Similarly, Monesha G et al. reported that 20% of patients with primary hypertension were born prematurely [1]. Previous studies consistently indicate that adults born prematurely or with low birth weight are at increased risk of developing hypertension and cardiovascular disease [20,21,22,23]. Research into children has also identified low birth weight as a significant risk factor for the development of high blood pressure [24].

Obesity plays a significant role in the development of primary hypertension. Our study found that the proportion of children with obesity is slightly higher in the primary hypertension group.

In our study, renal parenchymal disease was identified as the leading cause of secondary hypertension in 78 (68%) children, which is consistent with findings from previous studies [8]. Polycystic kidney disease (including ARPKD and nephronophthisis) accounted for one-third of renal causes, followed by congenital anomalies of the kidney and urinary tract (CAKUT). Similar results were reported in a study conducted in North America [24].

A study on chronic kidney disease (CKD) in Omani children found that hypertension was a common clinical feature at CKD presentation, observed in 85% of patients, particularly among those with glomerulonephritis and polycystic kidney disease compared to CAKUT [25].

The lower proportion of primary hypertension in our patient cohort may be attributed to the high number of patients with chronic kidney disease (CKD) included in our study. A previous study by Flynn et al. reported that 54% of children in a CKiD cohort either had hypertension or a history of hypertension [26]. Similarly, a study by Kramer et al. in Europe found that over two-thirds of children undergoing hemodialysis, peritoneal dialysis, or a kidney transplant were hypertensive [27].

Endocrine causes accounted for 10.6% (11 children) of secondary hypertension cases in our cohort, with two-thirds attributed to apparent mineralocorticoid excess. Data on the prevalence of endocrine hypertension in pediatric patients are limited, but in adults, it ranges between 5 and 10% [28].

Vascular causes were identified as the third etiology of secondary hypertension in 8.7% (nine children) of cases, with renal artery stenosis being the most frequent subtype. This finding is consistent with previous reports [29].

In this cohort, the most common complication observed was left ventricular hypertrophy (LVH), which was significantly more prevalent in children with secondary hypertension compared to those with primary hypertension (*p*-value = 0.019). Central nervous system complications, such as posterior reversible encephalopathy syndrome (PRES) and intracranial hemorrhage, were exclusively observed in children with secondary hypertension. Previous studies have consistently shown that children with secondary hypertension are more likely to exhibit target organ damage compared to those with primary hypertension, especially among children with chronic kidney disease (CKD) [10]. Additionally, research indicates that more than 40% of children diagnosed with primary hypertension already presented with LVH at the time of diagnosis [30,31].

Regarding management (Table 3), 19 children (14.7%) were managed with lifestyle modifications, primarily those with primary hypertension, while antihypertensive medications were predominantly used in cases of secondary hypertension. Approximately one-third of these patients required a combination of more than three medications to achieve blood pressure control. Calcium channel blockers were the most frequently prescribed class of medications, followed by beta-blockers, whereas angiotensin-converting enzyme inhibitors (ACE inhibitors, ACE-i) were utilized in only 21.7% of patients. The study by Uysal et al. indicated that 40.6% of patients were managed solely by lifestyle modifications, while 25.9% received calcium channel blockers, 9.5% were prescribed ACE inhibitors (ACE-i), 8.2% were treated with beta-blockers, and another 8.2% required two or more medications [10]. In another study involving 25 children with secondary hypertension, all of them received at least one antihypertensive medication, and 13% required a second antihypertensive medication [30].

Research by Yoon et al. on hypertensive adolescents aged 12–18 years revealed that the most commonly used antihypertensive medication was ACE inhibitors (ACE-i), and approximately 25% of patients required combination therapy with antihypertensive medications [32].

In this study, a total of 14 patients (13.6%) with secondary hypertension required surgical or radiological intervention to achieve blood pressure control (Table 3).

This study has several limitations that should be acknowledged. It is a retrospective cross-sectional review with a relatively small sample size, which may affect the statistical power of the study. The smaller number of cases in the primary hypertension group limited our ability to conduct robust comparisons and statistical analyses between both groups, necessitating caution in interpreting the results. Moreover, given that the majority of secondary hypertension cases stem from renal causes, this factor should be taken into account when analyzing specific findings, such as complication rates. Bias and confounders associated with renal disease may potentially influence the outcomes. Also, the assessment of cardiac function was carried out using a conventional transthoracic echocardiogram and not by speckle tracking echocardiography. Speckle tracking echocardiography, which is an innovative technique that measures myocardial deformation indices and allows for the detection of subclinical myocardial dysfunction, would have revealed incremental diagnostic and prognostic information on the myocardial structure and function of hypertensive children.

Additionally, the population displays significant skewness, with a mean age of 3.2 years. This characteristic could introduce bias in the assessment of secondary hypertension.

In conclusion, this study provides an overview of the demographic and clinical characteristics of children with hypertension. To our knowledge, it is the first study in the region to specifically address hypertension in children. We found that secondary hypertension was more common than primary hypertension among younger age groups. Hypertension in children is associated with significant morbidity and mortality. Therefore, early awareness and routine blood pressure screenings are essential for the early detection, management, and prevention of complications to improve patient outcomes.

## Figures and Tables

**Table 1:** Demographic data.

	Total	Primary Hypertension	Secondary Hypertension	*p*-Value
Number	129	26 (20.2%)	103 (79.8%)	0.001
Male/Female	73 (56.6%)/56 (43.4%)	18 (69%)/8	55 (53.4%)	0.082
Age at diagnosis Mean	3.2 ± 3.3			
Family History	65 (73.0%)	15 (57.7%)	50 (48.5%)	0.518
Consanguinity	77 (60.2%)	13 (50.0%)	64 (62.7%)	0.087
Prematurity	27 (20.9%)	6 (23.1%)	21 (20.6%)	0.528
Normal weight	53 (41%)	10 (38%)	43 (54%)	0.551
Underweight	38 (29%)	10 (38%)	28 (27%)	0.085
Overweight	11 (8.5%)	1 (4%)	10 (10%)	0.075
Obesity	14 (11%)	5 (19%)	9 (9%)	0.527

**Table 2:** Etiology of hypertension.

Etiology	Total (%)
Primary	26
Secondary	103
Renal	78 (60.5%)
CAKUT	21 (27%)
Nephrotic syndrome (SRNS)	14 (18%)
PKD (ARPKD and nephronophthisis)	24 (30.7%)
Glomuronephritis	11 (14%)
Wilms tumor	1 (1.2%)
Other	5 (6.4%)
Unknown	2 (2.6%)
Endocrine	11 (10.6%)
Vascular	9 (8.7%)
Medications	2 (3%)
Respiratory	2 (2%)
CNS	1 (0.97%)
Total	129 (100%)

Abbreviation: CAKUT, congenital anomalies of kidney and urinary tract; PKD, polycystic kidney disease; ARPKD, autosomal recessive polycystic kidney disease; CNS, central nervous system.

**Table 3:** Treatment and outcome.

Treatment	Primary Hypertension	Secondary Hypertension	*p*-Value
B-Blocker	3 (26%)	69 (67%)	0.001
Ca-Chanel blocker	11 (42%)	80 (78%)	0.001
ACE inhibitor	2 (7.7%)	26 (24.3%)	0.001
ARB	0	4 (3.9%)	0.44
A-Blocker	0	28 (27.2%)	0.002
Hydralazine	10 (38.5%)	66 (64%)	0.001
Diuretic	0	30 (29%)	0.001
Lifestyle change only	10 (38%)	9 (8.7%)	0.001
One medication	7 (26.9%)	9 (8.7%)	0.029
Two medications	7 (26.9%)	20 (19.4)	0.316
Three medications	2	27 (26%)	0.026
More than 3 medications	0	38 (36.9%)	0.001
Intervention	-	14 (13.6%)	
Renal angioplasty	-	2 (1.94%)	
Nephroblastoma removal	-	1 (1.0%)	
Nephrectomy	-	9 (8.7%)	
Coarctation of aorta repair	-	2 (1.9%)	
Complications			
Hypertensive urgency	2 (8%)	55 (53.4%)	0.001
Cardiovascular	5 (19.2%)	48 (47%)	0.019
Central nerves system	0	17 (16.5%)	0.030
PRES	0	15 (14.6%)	0.032
Intracranial hemorrhage	0	2 (1.94%)	0.373

Abbreviations: ACE, angiotensin converting enzyme inhibitor; ARB, angiotensin receptor blocker; PRES, posterior reversible encephalopathy syndrome.

## Data Availability

The data are not publicly accessible due to patient privacy considerations; however, they can be obtained from the corresponding author upon reasonable request.
